# Molecular Mechanisms Responsible for Pharmacological Effects of Genipin on Mitochondrial Proteins

**DOI:** 10.1016/j.bpj.2019.10.021

**Published:** 2019-10-24

**Authors:** Jürgen Kreiter, Anne Rupprecht, Lars Zimmermann, Michael Moschinger, Tatyana I. Rokitskaya, Yuri N. Antonenko, Lars Gille, Maria Fedorova, Elena E. Pohl

**Affiliations:** 1 Institute of Physiology, Pathophysiology and Biophysics, Department of Biomedical Sciences, University of Veterinary Medicine, Vienna, Austria; 2 Rostock University Medical Center, Rostock, Mecklenburg-Vorpommern, Germany; 3 Belozersky Institute of Physico-Chemical Biology, Lomonosov Moscow State University, Moscow, Russia; 4 Institute of Pharmacology and Toxicology, Department of Biomedical Sciences, University of Veterinary Medicine, Vienna, Austria; 5 Institute of Bioanalytical Chemistry, Faculty of Chemistry and Mineralogy, Center for Biotechnology and Biomedicine, University of Leipzig, Leipzig, Germany

## Abstract

Genipin, a natural compound from *Gardenia jasminoides*, is a well-known compound in Chinese medicine that is used for the treatment of cancer, inflammation, and diabetes. The use of genipin in classical medicine is hindered because of its unknown molecular mechanisms of action apart from its strong cross-linking ability. Genipin is increasingly applied as a specific inhibitor of proton transport mediated by mitochondrial uncoupling protein 2 (UCP2). However, its specificity for UCP2 is questionable, and the underlying mechanism behind its action is unknown. Here, we investigated the effect of genipin in different systems, including neuroblastoma cells, isolated mitochondria, isolated mitochondrial proteins, and planar lipid bilayer membranes reconstituted with recombinant proteins. We revealed that genipin activated dicarboxylate carrier and decreased the activity of UCP1, UCP3, and complex III of the respiratory chain alongside with UCP2 inhibition. Based on competitive inhibition experiments, the use of amino acid blockers, and site-directed mutagenesis of UCP1, we propose a mechanism of genipin’s action on UCPs. At low concentrations, genipin binds to arginine residues located in the UCP funnel, which leads to a decrease in UCP’s proton transporting function in the presence of long chain fatty acids. At concentrations above 200 *μ*M, the inhibitory action of genipin on UCPs is overlaid by increased nonspecific membrane conductance due to the formation of protein-genipin aggregates. Understanding the concentration-dependent mechanism of genipin action in cells will allow its targeted application as a drug in the above-mentioned diseases.

## Significance

Genipin is a well-known natural cross-linking agent for proteins, collagen, gelatin, and chitosan. However, the mechanism of its multiple effects on cells and mitochondria is under dispute. Mitochondrial UCP2 was previously revealed as an important target for genipin. We show that inhibition of UCP2 by genipin at submillimolar concentrations depends on the presence of three positively charged arginines in the funnel of the protein. This mechanism is similar to the UCP inhibition by purine nucleotides such as ATP/ADP and GTP/GDP. The effect of genipin is not specific to UCP2, which can be explained by the presence of arginines in homologous UCP1 and UCP3. This insight is crucial for the design of specific inhibitors of UCPs.

## Introduction

**Figure 1:**
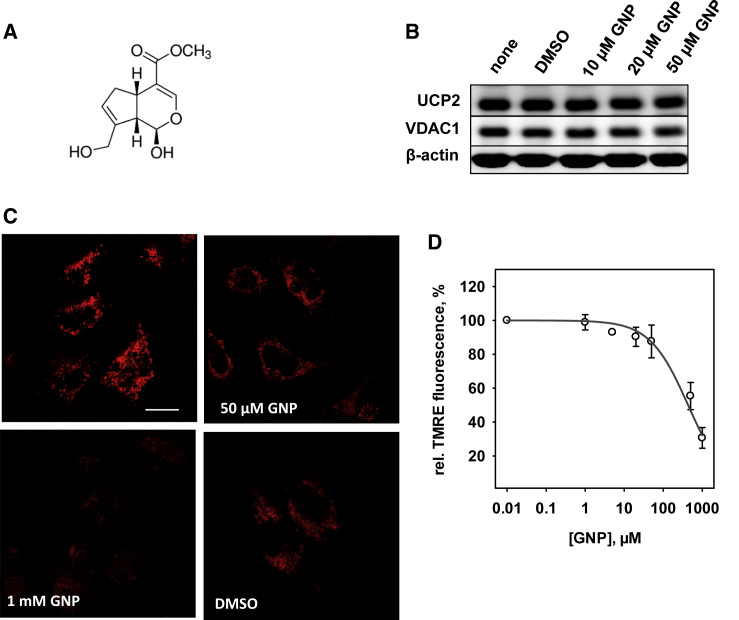
Effect of genipin on UCP2-expressing neuroblastoma cells N18TG2. (*A*) The chemical structure of genipin. (*B*) Western blot analysis of UCP2 in N18TG2 cells after 24 h incubation with 10, 20, and 50 *μ*M genipin (GNP) dissolved in DMSO, DMSO as vehicle, and without treatment (control). VDAC1 and *β*-actin were used as controls for mitochondrial number and protein loading, respectively. (*C*) Fluorescence microscopy images of N18TG2 cells loaded with TMRE (*red*) as a mitochondrial membrane potential indicator. Top left: control without genipin; top right: after 1 h incubation with 50 *μ*M genipin; bottom left: after 1 h incubation with 1 mM genipin is shown; and bottom right: after 1 h incubation with DMSO as vehicle. Scale bars represent 20 *μ*m. (*D*) Dependence of the TMRE fluorescence signal intensity on genipin (GNP) concentration in N18TG2 cells. Data are normalized to the control (DMSO) in the absence of genipin and fitted with a four-parameter logistic function (Eq. 2). Data are the means ± SD of three independent experiments.

Genipin is an aglycone of iridoid glycoside called geniposide (Fig. 1 *A*). It is mainly found in the fruits of *Gardenia jasminoides* and *Gardenia americana* (1,2). In traditional Chinese medicine, genipin is widely used to treat cancer (3, 4, 5), inflammation (6, 7, 8), diabetes (9,10), and neurological disorders (11,12). However, genipin is still not popular in classical medicine, mainly because its mechanism of action is poorly understood. Genipin is usually described as a strong cross-linker with less cytotoxicity compared to glutaraldehyde (13, 14, 15, 16). A cross-linking mechanism is believed to occur between genipin and molecules containing primary amines (17). Interestingly, genipin was reported to specifically inhibit uncoupling protein 2 (UCP2) in the mitochondria of isolated pancreatic islets (18) by an unknown mechanism that is unrelated to cross-linking.

UCP2 is a member of the mitochondrial anion transporter superfamily (19) and is highly homologous to other members of the UCP subfamily, such as UCP3 and UCP1 (20). It has been identified in rapidly proliferating cells that rely on glycolysis, such as stem cells (21,22), activated immunological cells (23), cancer cells, and immortalized cell lines (22,24,25). UCP2 mediates a regulated proton leak through the inner mitochondrial membrane and was recently suggested to transport small C4 metabolites (26). Based on its different transport functions, UCP2 has been implicated in the regulation of ROS (27) and/or metabolic adaptation by the facilitation of glutamine utilization (28).

UCP-mediated proton transport is activated by long chain fatty acids (FA) and inhibited by purine nucleotides (29, 30, 31, 32). According to the FA cycling model (33, 34, 35), protons are transported by the neutral form of FA (flip-flop (36)). UCP2 (similar to UCP1 and UCP3) facilitates the transport of the FA anionic form through the membrane. Although genipin is increasingly used for selective inhibition of UCP2 in living cells and isolated mitochondria, there is reason to believe that its action is not only limited to UCP2. However, multiscale systems provide only limited information about genipin specificity. The goal of this work was to evaluate the action of genipin on various mitochondrial (UCP1, UCP2, UCP3, dicarboxylate carrier (DIC), and complex III (CIII)) and nonmitochondrial (*α*-hemolysin (*α*HL)) proteins. We employ neuroblastoma cells and mitochondria to analyze the impact of genipin on cellular, mitochondrial, and protein expression levels. Further, we evaluated the concentration-dependent effects of genipin in the well-defined system of bilayer membranes reconstituted with recombinant UCPs. Based on competitive inhibition experiments, site-directed mutagenesis, and MS analysis, we propose a mechanism by which genipin at low concentrations leads, among other effects, to the inhibition of UCP-mediated proton leak.

## Materials and Methods

### Chemicals

KCl, Na_2_SO_4_, 2-(N-morpholino)ethanesulfonic acid (MES), tris(hydroxymethyl)aminomethane (Tris), EGTA, hexane, hexadecane, cytochrome c (Cyt c), decylubiquinone, KCN, sucrose, 3-(N-morpholino)propanesulfonic acid (MOPS), bovine serum albumin (BSA), arachidonic acid (AA), genipin, geniposide, adenine triphosphate (ATP), ammonium phosphate (monobasic, NH_4_H_2_PO_4_), dimethyl sulfoxide (DMSO), sulfo-NHS-acetate (NHS), methyl-4-nitrobenzenesulfonate (MNBS), N-ethylmaleimide (NEM), and diammonium salt of malic acid were purchased from Sigma-Aldrich (Munich, Germany). EDTA, KH_2_PO_4_, NaN_3_, and acetonitrile were purchased from Merck (Darmstadt, Germany). Diphytanoylphosphatidylcholine, 1,2-dioleoyl-*sn*-glycero-3-phosphocholine (DOPC), 1,2-dioleoyl-*sn*-glycero-3-phosphoethanolamine (DOPE), and cardiolipin (CL) came from Avanti Polar Lipids (Alabaster, AL). Chloroform was obtained from Carl Roth (Karlsruhe, Germany).

### Cloning, mutation, and expression of UCPs

Mouse UCP1, UCP2, and UCP3 were cloned and expressed as described in (37,38). Single mutants were generated as described in (39). To prepare double and triple mutants, mutations were inserted one after another. Wild-type (wt) and mutant UCPs were expressed in *Escherichia coli* strain Rosetta.

### Measurements of electrical parameters of membranes reconstituted with UCPs or *α*HL

Recombinant UCP1–UCP3 were purified from *E. coli* inclusion bodies and reconstituted into liposomes as described in (39). The purity of the recombinant proteins is shown in Fig. S1. AA at a concentration of 15 mol% was directly added to the lipid phase before membrane formation. Buffer contained 50 mM Na_2_SO_4_, 10 mM Tris, 10 mM MES, and 0.6 mM EGTA at pH = 7.34. Planar lipid bilayers were formed from proteoliposomes or liposomes on the tips of plastic pipettes, as described previously (40). Proper membrane formation was verified by measuring membrane capacitance (C = 0.72 ± 0.05 *μ*F/cm^2^). Protein, FA, and genipin did not affect membrane capacity. Current-voltage measurements were performed with a patch-clamp amplifier (EPC 10 USB; HEKA Elektronik Dr. Schulze, Lambrecht, Germany). Total membrane conductance (G) at 0 mV was obtained as a slope of a linear fit of experimental data at applied voltages from −50 to +50 mV. Genipin was dissolved in DMSO or in water, and ATP was dissolved in water. Both were added to the buffer solution before the membrane formation in concentrations indicated in figure descriptions. Incubation time was 30 min at 33°C. We performed a control measurement showing that DMSO has no effect on the membrane conductance if added up to volume 10 *μ*L per 750 *μ*L buffer solution (Fig. S2).

In experiments with pH gradient (Genipin at high concentrations increases membrane conductance by nonspecific ion transport), the adjustment of the pH value was done in the presence of a stable bilayer membrane by adding 10 *μ*L of a 10 mM Tris solution to the buffer solution while stirring. Thus, the pH value in the plastic tip remained unchanged, and pH values in bulk were measured before and after the measurement. Because we used Tris to change pH value, a salt asymmetry can be excluded (41). The solution was stirred for at least 20 s before measurement.

Membrane conductance in relative units, *G*_*rel*_, was calculated according to Eq.1:

(1) $$G_{\mathrm{rel}}=\frac{G-G_{\mathrm{AA}}}{G_{0}-G_{\mathrm{AA}}},$$

where *G*_*0*_ depicts the conductance of membrane reconstituted with UCPs and AA without genipin, *G* depicts the conductance of membrane reconstituted with UCPs and AA after the addition of genipin, and *G*_*AA*_ depicts the conductance of a membrane reconstituted with 15 mol% AA in the absence of UCPs and genipin. Half maximal inhibitory concentration (IC_50_) values for proteins were obtained by the regression fit of a four-parameter logistic function (Eq. 2) to the experimental data, as follows:

(2) $$y=d+\frac{a-d}{1+{\left(\frac{x}{c}\right)}^{b}},$$

where *x* is the concentration of inhibitor, *y* is the relative conductance, *d* is the remaining relative conductance at infinite inhibitor concentration, *a* is the relative conductance in the absence of inhibitor, *c* is the IC_50_ value, and *b* is the Hill’s slope of the curve. The data in Fig. 6 *A* were fitted by Eq. 3, which is the sum of two logistic functions:

(3) $$y=d+h-a+\frac{a-d}{1+{\left(\frac{x}{c}\right)}^{b}}+\frac{a-h}{1+{\left(\frac{x}{g}\right)}^{f}},$$

where *x* is the genipin concentration, and *y* is the relative conductance. Parameters *a*, *b*, *c*, and *d* correspond to the parameters in Eq. 2. Parameter *g* is the half-effective concentration, *h* is the maximal conductance at an infinite genipin concentration, and *f* is the Hill’s slope of the activation by genipin.

For the electrophysiological characterization of *α*HL, black lipid membranes (BLM (42)) were formed in a hole of a Teflon partition (0.25-mm diameter) separating two compartments of a measurement chamber. Membranes were made from a 2% solution of diphytanoylphosphatidylcholine in n-decane. Buffer solution was made of 1 M KCl, 10 mM Tris, and 10 mM MES at pH = 7.5. The experiments were carried out at room temperature (23–25°C). *α*HL was a generous gift of Dr. Pavel Nazarov (Moscow State University, Moscow, Russia) and was prepared as described previously (43). Small amounts of *α*HL were added on the *cis* side of the chamber (the side connected to the ground electrode). Spontaneous channel insertion was obtained while stirring under applied voltage. Voltage was applied to BLMs with Ag/AgCl electrodes placed into the solutions on the two sides of the BLM and connected via agar bridges. The electric current was recorded under voltage clamp conditions using a patch-clamp amplifier (model BC-525C; Warner Instruments, Hamden, CT). Signals were filtered by a low pass Bessel filter at 500 Hz and digitized using an NI-DAQmx (National Instruments, Austin, TX) with a sampling frequency of 10 kHz. Single channel analysis was performed using WinEDR Strathclyde Electrophysiology Software designed by J. Dempster (University of Strathclyde, Glasgow, UK).

### Determination of mitochondrial CIII activity

Decylubiquinol (dUQH_2_) was prepared from decylubiquinone, and the isolation of mitochondrial CIII from bovine heart and enzyme inhibition measurements followed a published protocol (44). The enzyme was suspended in 1 mL of buffer (pH 7.2, 25°C) containing 250 mM sucrose, 50 mM KH_2_PO_4_, 0.2 mM EDTA, 2.5 mM KCN, 2 mM NaN_3_, and 100 *μ*M cyt c^3+^. Genipin dissolved in water or antimycin A (AmA) dissolved in acetonitrile were incubated for 30 min at 25°C with the reaction mixture. After the addition of 75 *μ*M dUQH_2_, enzymatic reduction of cyt c^3+^ to cyt c^2+^ was measured by spectrophotometric analysis of the absorbance at 550 nm (cyt c^2+^) compared to 540 nm (isosbestic point) using a Shimadzu Multispec 1501 diode array photometer. Enzymatic activity was obtained by the slope of a linear fit to the data within the first 30 s after the addition of dUQH_2_ using an extinction coefficient of *ε*_550–540 nm_ = 19 mM^−1^ cm^−1^ for cyt c^2+^ (45). Activity was corrected for the chemical reduction of cyt c^3+^ in the absence of CIII. The enzymatic activity R of CIII is related to the noninhibited activity R_0,_ which are both corrected for the activity in the absence of CIII, R_C_ (R_rel,_ Eq. 4), as follows:

(4) $${\text{R}}_{\text{rel}}=\frac{\text{R}-{\text{R}}_{\text{C}}}{{\text{R}}_{0}-{\text{R}}_{\text{C}}}.$$

The level of Cyt c reduction, R_C_, was the same in the absence or presence of genipin.

### Isolation and measurements of the activity of mitochondrial DIC

DIC activity was assayed by the mitochondrial swelling in ammonium malate medium as previously described (46). Liver mitochondria were isolated from rats by differential centrifugation in medium containing 250 mM sucrose, 10 mM MOPS, and 1 mM EGTA at pH 7.4 (47). The final washing was performed in medium that also contained BSA (0.1 mg/mL). The mitochondrial protein concentration (0.5 mg/mL) was determined using the Biuret method. The absorbance of the mitochondrial suspension was recorded at 600 nm by Amersham Pharmacia Ultrospec 1100 Pro UV/Vis spectrophotometer. The incubation medium was 100 mM ammonium malate, 10 mM Tris, 0.2 mM EDTA, and 3 *μ*M rotenone at pH 7.4. Swelling was initiated by the addition of 10 mM ammonium phosphate. Octyl-malonate (OM), an inhibitor of DIC, was a generous gift of Dr D. I. Bondarenko (Bach Institute of Biochemistry, Moscow, Russia).

Animal use and experimental procedures were conducted in accordance with the international guidelines for animal care and use and were approved by the Institutional Ethics Committee of A.N. Belozersky Institute of Physico-Chemical Biology at Moscow State University.

### Cell culture

N18TG2 cells (Deutsche Sammlung von Mikroorganismen und Zellkulturen, Braunschweig, Germany) were cultivated at 37°C and 5% CO_2_ as described previously (48). Cell culture medium comprised Dulbecco’s modified Eagle medium (DMEM; 21.6 mM glucose) supplemented with 9.6% fetal bovine serum, 3.85 mM glutamine, and 1.92 mM sodium pyruvate (all obtained from Sigma-Aldrich). Before beginning measurements, cells were seeded in four-well Petri dishes (Greiner Bio-One, Germany), coated with poly-D-lysine (Sigma-Aldrich) with 0.5 mL medium per well and were cultured for 24–72 h.

### Confocal microscopy

Potential-sensitive dye tetramethylrhodamine, ethyl ester (TMRE; purchased from Sigma-Aldrich) was added to the cells 20 min before the start of the experiment at a final concentration of 12.5 nM. Fluorescence was excited at wavelength 561 nm with a DPPS laser and measured with an inverse confocal laser scanning microscope (TCS SP5 II; Leica Microsystems, Wetzlar, Germany). The latter was equipped with a heating box to maintain a temperature of 37°C and with a 5% CO_2_ supply (48), which allowed long-term measurements using living cells. Fluorescence was collected through a 63× water or 40× oil immersion objectives in an emission channel of 570–690 nm. *Z*-stacks of cells with a step size of 500 nm (256 × 256 pixels; 400 Hz; 73 frames per *z*-stack) were recorded every 3 min for typically 1 h.

### Silver staining

15 *μ*L UCP1 proteoliposomes (protein concentration was 63.1 *μ*g/mL, internal charge was 84) were incubated with 10, 50, 100, 200, 500, and 1000 *μ*M genipin and an appropriate amount of water as a vehicle for 30 min at 37°C. Proteins were degraded at 97°C for 10 min in sodium dodecyl sulfate (SDS) sample buffer (pH 6.0) containing 25 mM Tris, 2.5% glycerin, 1% SDS, 1% *β*-mercaptoethanol, and bromophenol blue. Samples and protein ladder (Bio-Rad Laboratories, Hercules, CA) were loaded on 15% SDS gels. Electrophoresis was performed at 120 mV for 3 h. Silver staining of the gel was performed following a standard protocol.

### Western blot analysis

The collection of total cellular protein from cell culture samples and western blot analysis for UCP2 was performed as described previously (23). For all western blot analyses, 20 *μ*g total protein per lane was loaded onto 15% SDS gels. Affinity-purified polyclonal antibody directed against murine UCP2 was originated from our own laboratory (23) and was used at a dilution of 1:1000. To verify protein loading and detect the proteins of interest, the following antibodies were used (dilution in parentheses): voltage-dependent anion channel, anti-VDAC1 (ab14734; 1:5000; Abcam, Cambridge, UK), and anti-*β*-actin (A5441; 1:5000; Sigma-Aldrich). For that, membranes were stripped and reblocked before incubation with diluted antibody in blocking solution.

### Mass spectrometry

Liposomes were made of either DOPC:CL (90:10 mol%) or DOPE:DOPC:CL (45:45:10 mol%). Liposomes were incubated with 50 *μ*M or 1 mM genipin in buffer solution, which are the same concentrations that were used for electrophysiological measurements. Liposomes were mixed with chloroform/methanol (1:1, v/v). An organic phase containing modified lipids was separated by centrifugation (4000 × *g*, 10 min, room temperature), diluted 1:5 (v/v) in ESI solution (methanol: chloroform (2:1 v/v) containing 5 mM ammonium formate), and analyzed by direct injection using robotic nanoflow ion source TriVersa NanoMate (Advion, Ithaca, NY). The latter was equipped with nanoelectrospray chips (1.5 kV ionization voltage, 0.4 psi back pressure) coupled to a LTQ Orbitrap XL ETD mass spectrometer (Thermo Fisher Scientific, Bremen, Germany). The temperature of the transfer capillary was set to 200°C, and the tube lens voltage was set to 115 V. Mass spectra were recorded from *m/z* of 400–2000 in the Orbitrap mass analyzer at a mass resolution of 100,000 at *m/z* 400. Tandem mass spectra were acquired by performing collision-induced dissociation (CID; isolation width 1–1.5 units, normalized collision energy 25–30%, activation time 30 ms, activation Q 0.25) in the linear ion trap. Data were acquired and analyzed using Xcalibur software (version 2.0.). All tandem MS spectra were manually annotated.

To analyze UCP1 modifications, proteins separated by SDS-PAGE and stained with Coomassie blue were excised, destained with acetonitrile (50%, v/v, in 50 mM NH_4_HCO_3;_ 1 h, 37°C, 750 rpm), dehydrated with 100% acetonitrile, and dried by vacuum. Proteins were digested with trypsin (375 ng in 3 mM in NH_4_HCO_3_, 4 h, 37°C, 550 rpm), and peptides were extracted using consecutive incubations with 100, 50, and 100% acetonitrile (15-min sonication for each step). Combined extracts were vacuum concentrated and stored at −20°C. Before MS analysis, peptides were dissolved in 10 *μ*L 60% aqueous acetonitrile containing 0.5% formic acid and further diluted 1:20 with 3% aqueous acetonitrile. A nanoAcquity UPLC (Waters, Eschborn, Germany) was coupled online to an LTQ Orbitrap XL ETD mass spectrometer equipped with a nano-ESI source (Thermo Fisher Scientific). Eluent A was aqueous formic acid (0.1%, v/v), and eluent B was formic acid in acetonitrile (0.1%, v/v). Samples (10 *μ*L) were loaded onto the trap column (nanoAcquity Symmetry C18, internal diameter 180 *μ*m, length 20 mm, particle diameter 5 *μ*m) at a flow rate of 10 *μ*L/min. Peptides were separated on BEH 130 column (C18-phase, internal diameter 75 *μ*m, length 100 mm, particle diameter 1.7 *μ*m) with a flow rate of 0.4 *μ*L/min using two step gradients from 3 to 30% eluent B over 18 min and then to 85% eluent B over 1 min. After an equilibration time of 12 min, samples were injected every 33 min.

The transfer capillary temperature was set to 200°C, and the tube lens voltage was set to 120 V. An ion spray voltage of 1.5 kV was applied to a PicoTip online nano-ESI emitter (New Objective, Berlin, Germany). The precursor ion survey scans were acquired at an Orbitrap (resolution of 60,000 at *m/z* 400) for a *m/z* range from 400 to 2000. CID-tandem mass spectra (isolation width 2, activation Q 0.25, normalized collision energy 35%, activation time 30 ms) were recorded in the linear ion trap by data-dependent acquisition for the top six most abundant ions in each survey scan with dynamic exclusion for 60 s using Xcalibur software (version 2.0.7). Peptides were identified using the SEQUEST search engine (Proteome Discoverer 1.4; Thermo Fisher Scientific) against the UniProt database, allowing up to two missed cleavages and a mass tolerance of 10 ppm for precursor ions and 0.8 Da for product ions. The results were filtered for rank 1 high confidence peptides and score versus charge states corresponding to Xcorr/z 2.0/2, 2.25/3, 2.5/4, and 2.75/5.

### Statistical analysis

Data are displayed as the mean ± SD of at least three independent measurements.

## Results

### Genipin does not influence UCP2 expression but decreases the mitochondrial membrane potential (*Φ*_m_) of neuroblastoma cells at high concentrations

It was previously shown that neuroblastoma cell line N18TG2 expresses UCP2 (22). To determine whether genipin (Fig. 1 *A*) affects UCP2 protein levels, we incubated N18TG2 cells with 10, 20, and 50 *μ*M genipin for 24 h and performed western blot analysis using anti-UCP2 antibody. Fig. 1 *B* shows that UCP2 expression is not regulated by genipin, which is in contrast to previous reports (9).

Next, we tested whether genipin affects mitochondrial potential, *Φ*_*m*_, in neuroblastoma cells. Genipin at concentrations 50 *μ*M and 1 mM was added to cells stained with potential-sensitive dye TMRE. The intensity of the fluorescence signal was measured before (I_0_) and each 3 min during 1 h after genipin addition (I_t_). I_rel_ (I_t_/I_0_^∗^100%) only slightly decreased after the addition of 50 *μ*M genipin (Fig. 1 *C*, *top right*; Fig. 1 *D*), whereas in the presence of 1 mM genipin, I_rel_ was reduced by 70%, which corresponds to a strong reduction of *Φ*_m_ (Fig. 1 *C*, *bottom left*; Fig. 1 *D*). By fitting the data to a four-parameter logistic function (Eq. 2), we obtained an IC_50_ of 456 ± 73 *μ*M. However, the decrease in *Φ*_m_ is difficult to explain by alteration in UCP2 expression or activity because a strong cross-linking effect of genipin at this high concentration might affect the cell viability.

### Genipin decreases the conductance of membranes reconstituted with UCPs

**Figure 2:**
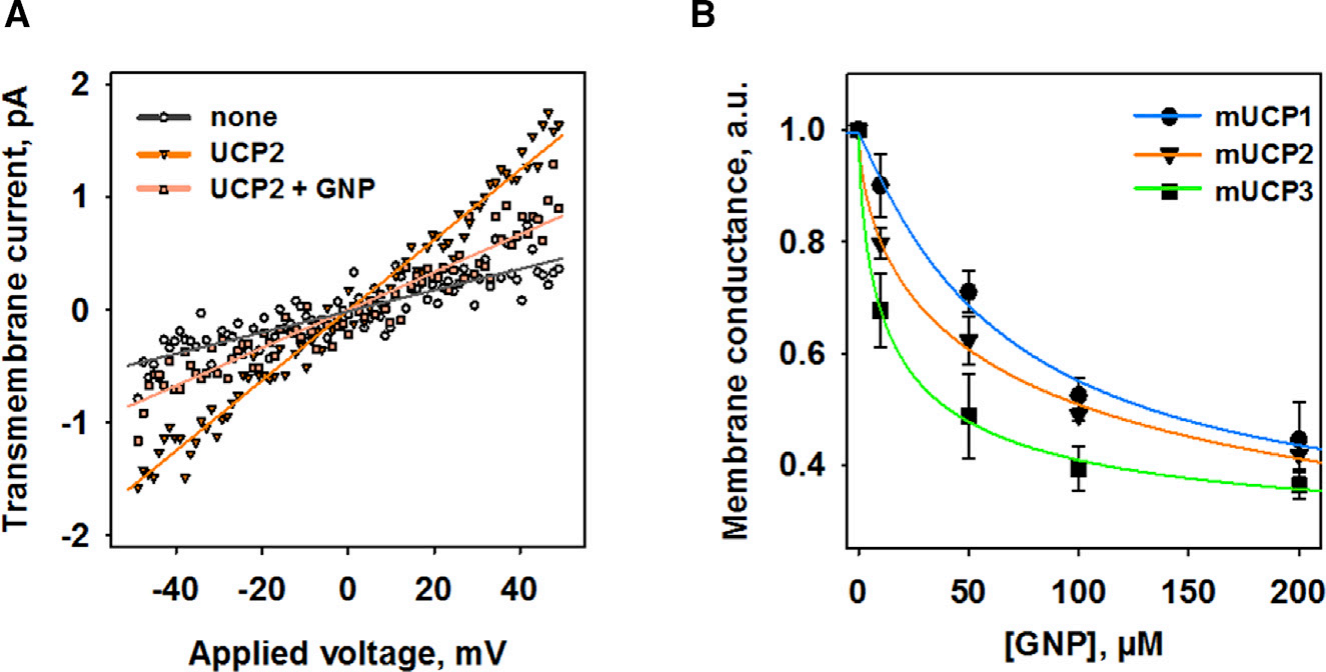
Inhibition of the proton transport activity of mitochondrial UCPs by genipin. (*A*) Representative current-Voltage measurements of membranes reconstituted with UCP2 in the absence (▾) and presence (▪) of 50 *μ*M genipin (GNP). The control (none) is the current mediated only by arachidonic acid (AA) in the absence of mUCP2. The lines represent a linear fit to the curves. The lipid bilayer membranes were made of 45:45:10 mol%:DOPE:CL. 15 mol% of AA was added to the lipid phase before membrane formation. The concentrations of lipid and UCP2 were 1.5 mg/mL and 6 *μ*g per mg of lipid, respectively. The buffer solution contained 50 mM Na_2_O_4,_ 10 mM Tris, 10 mM MES, and 0.6 mM EGTA at pH = 7.34 and T = 33°C. (*B*) Effect of GNP on the total conductance of membranes reconstituted with UCP1, UCP2, and UCP3. The relative conductance is the ratio of the G_0_ and G in the absence and presence of genipin (Eq. 1). The lines represent a regression fit of a four-parameter logistic function (Eq. 2) to the data. The data points are the means ± SD of three independent experiments. The experimental conditions are similar to those in (*A*). To see this figure in color, go online.

We further tested whether genipin affected the protonophoric function of recombinant UCPs reconstituted in planar lipid bilayer membranes. We started with UCP2, for which genipin has been described as a specific inhibitor based on experiments with knockout mice (18). Fig. 2 *A* shows that the slope of the current-voltage curve is increased in the presence of UCP2, activated by polyunsaturated long chain AA, and decreased after the addition of genipin. These findings correspond to a decrease in total membrane conductance (G) and UCP2-mediated proton transport.

We further evaluated the G of membranes reconstituted with UCP1, UCP2, and UCP3 at genipin concentrations from 0 to 200 *μ*M. Fig. 2 *B* and Fig. S3 demonstrate a decrease in G in a concentration-dependent manner with equal maximum inhibition for all three proteins. The IC_50_ values, calculated from fitting Eq. 2 to the experimental data, increased in the order UCP3 (41 ± 12 *μ*M) < UCP2 (94 ± 24 *μ*M) < UCP1 (133 ± 12 *μ*M).

### Genipin reduces the activity of CIII

**Figure 3:**
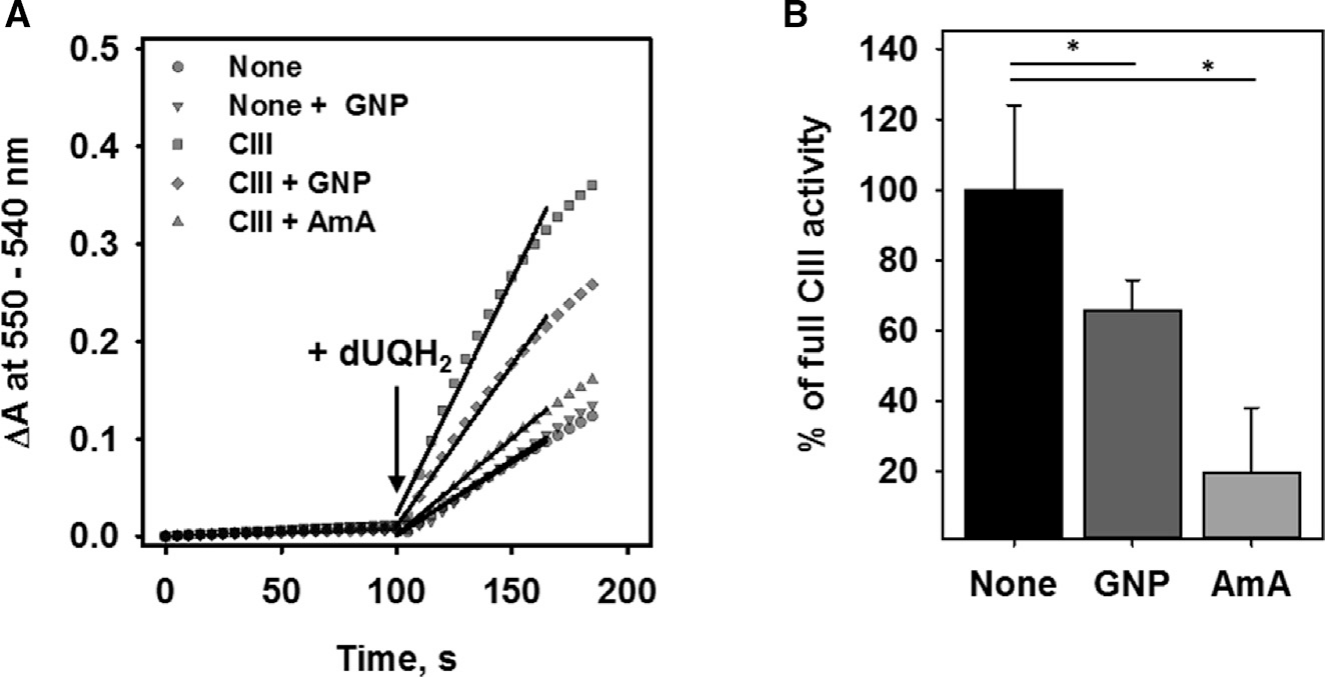
Effect of genipin on the activity of mitochondrial CIII. (*A*) Time course of the absorbance by reduction of cytochrome c (Cyt c) of CIII in the absence of inhibitors (▪) and in the presence of 20 *μ*M antimycin A (AmA, ▾) or 50 *μ*M genipin (GNP, ♦) added directly to buffer solution 30 min before measurement. The control experiments include the reduction of Cyt c in the absence of CIII (●) and in the presence of GNP without CIII (▪). The concentrations of protein, Cyt c, and decylubiquinone (dUQH_2_) were 12 nM, 100 *μ*M, and 75 *μ*M, respectively. The buffer contained 250 mM sucrose, 50 mM KH_2_O_4_, 0.2 mM EDTA, 2.5 mM KCN, and 2 mM NaN_3_ at pH = 7.2 and T = 33°C. The enzymatic activity was obtained by linear fit to the data within 30 s after the addition of dUQH2 at T = 100 s as indicated by the arrow. (*B*) CIII activity upon the addition of 50 *μ*M GNP (*second bar*) or 20 *μ*M AmA (*third bar*) compared to the maximal CIII activity (*first bar*). The results are means ± SD of at least three independent measurements. Significance was tested using paired *t*-test (^∗^*p* < 0.05).

The effects that were described to be specific for UCP2 (18) could in fact also have been explained by a decreased activity of the respiratory chain proteins. We next evaluated whether genipin affects the activity of CIII (Fig. 3 *A*). The activity of CIII was reduced to 63 ± 7% (Fig. 3 *B*; *second bar*) in the presence of 50 *μ*M genipin compared to the untreated control (Fig. 3 *B*; *first bar*). The addition of 20 *μ*M antimycin A (AmA) as a positive control decreased the CIII activity to 25 ± 17% (Fig. 3 *B*; *third bar*).

### Genipin activates mitochondrial DIC

**Figure 4:**
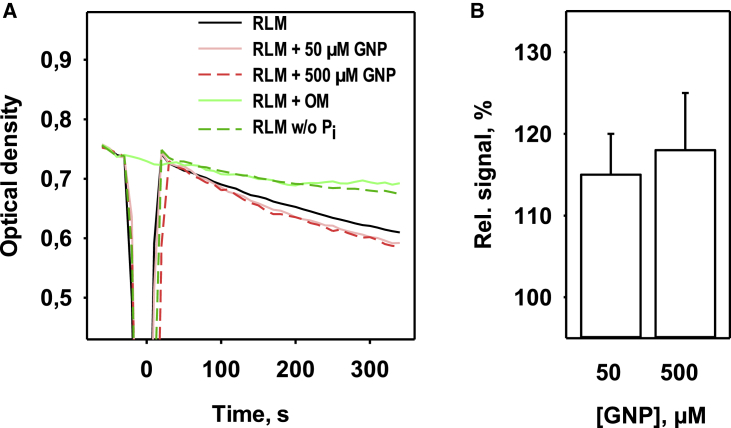
Effect of genipin on the activity of the mitochondrial dicarboxylate carrier (DIC). (*A*) Time course of the optical density measured at 600 nm in isolated rat liver mitochondria (RLM) in buffer medium (*black line*, control) in the presence of genipin (50 *μ*M (*light red*), 500 *μ*M (*dark red*, *dashed line*)) or octyl-malonate (OM, *dark green*, *dashed line*) and in the absence of phosphate (*light green*, *solid line*). Phosphate was added at t = 0 s. (*B*) Increase in the swelling rate of mitochondria in the presence of 50 and 500 *μ*M genipin (GNP) compared to the untreated control. The buffer medium contained 100 mM ammonium malate, 10 mM Tris, 0.2 mM EDTA, and 3 *μ*M rotenone (pH = 7.4, at room temperature). The data are the means ± SD of three independent measurements. To see this figure in color, go online.

DIC catalyzes the transport of dicarboxylates (malonate, malate, and succinate) across the inner mitochondrial membrane in exchange for inorganic anions (phosphate, sulfate, and thiosulfate). DIC activity was measured by mitochondrial swelling in ammonium malate medium after the addition of ammonium phosphate at t = 0 s (Fig. 4 *A*).

The addition of genipin to isolated rat liver mitochondria before phosphate led to a more rapid decrease in OD600 compared to the control, which indicates the enhanced mitochondrial swelling (Fig. 4 *B*). Mitochondrial swelling rates were 115 ± 5% (Fig. 4 *B*, *first bar*) and 118 ± 7% (Fig. 4 *B*, *second bar*) in the presence of 50 and 500 *μ*M genipin, respectively, compared to the control in the absence of genipin (100%).

We performed additional experiments and found that OM, which inhibited swelling in the presence and absence of genipin, was at the same level (see Fig. S4). Therefore, genipin activates swelling in a specific way mediated by activation of DIC.

### Genipin does not change the ion current mediated by *α*HL

**Figure 5:**
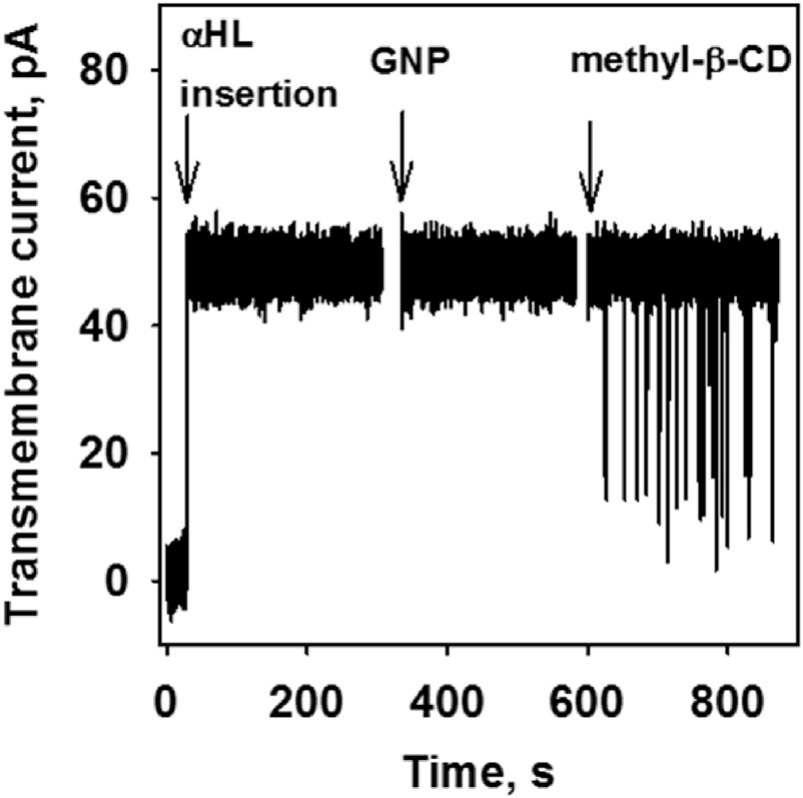
Channel activity of *α*HL in the presence and absence of genipin (GNP). The first arrow indicates the insertion of *α*HL, which results in a current around 50 pA at an applied potential of 50 mV. The second arrow indicates the addition of 50 *μ*M GNP to the buffer solution. The addition of 50 *μ*M methyl-*β*-cyclodextrin (CD) is indicated by the third arrow. The buffer solution contained 1 M KCl, 10 mM Tris, and 10 mM MES at pH = 7.5 and T = 23–25°C.

In the next step, we evaluated the effect of genipin on the nonmitochondrial *β*-barrel protein *α*HL. Fig. 5 shows that after protein insertion into the membrane, a transmembrane pore was formed. Genipin did not affect the ion channel current in contrast to methyl-*β*-cyclodextrin, which is a known inhibitor of *α*HL (49).

### Genipin at high concentrations increases membrane conductance by nonspecific ion transport

**Figure 6:**
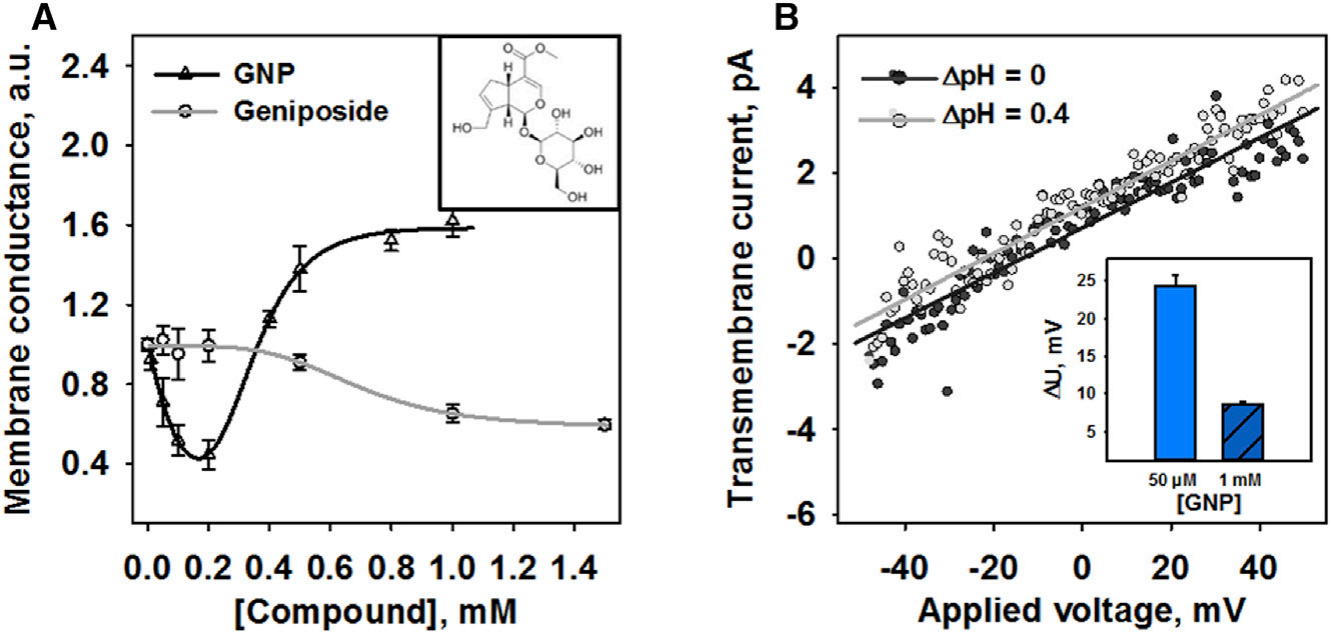
High genipin concentrations increase membrane conductance by nonspecific ion transport through the membrane. (*A*) Effect of genipin (Δ) and geniposide (○) on the total membrane conductance reconstituted with UCP1 relative to uninhibited UCP1 activity. The *x* axis denotes the concentration of either genipin or geniposide. The geniposide and genipin data were fitted with Eqs. 2 and 3, respectively. Inset: chemical structure of geniposide. (*B*) Representative current-voltage characteristics of membranes reconstituted with UCP1 in the presence or absence of a transmembrane pH gradient (0.4) and in the presence of 1 mM genipin. The lines represent a linear fit to the data in the range of −50 to +50 mV, from which the voltage shift at the *x* axis intercept (I = 0) was calculated. Insert: voltage shift in the presence of 50 *μ*M (*first bar*) and 1 mM GNP (*second bar*). In all measurements, membranes were made of 45:45:10 mol% phosphocholine (PC):PE:CL and 15 mol% AA added to the lipid phase before the membrane formation. The buffer solution contained 50 mM Na_2_O_4_, 10 mM Tris, 10 mM MES, and 0.6 mM EGTA at pH = 7.34 and T = 33°C. The membrane lipid and protein concentrations were 1.5 mg/mL and 4 *μ*g/(mg lipid), respectively. The data are the means ± SD of three independent measurements. To see this figure in color, go online.

To get more insight into the molecular mechanism of UCP inhibition by genipin, we applied an iridoid glycoside geniposide (Fig. 6 *A*, *inset*) and its metabolite genipin at concentrations up to 1 mM to UCP1, which was comprehensively characterized previously (39,50). Fig. 6 *A* demonstrates that inhibition by genipin stops at around 200 *μ*M and is superimposed by a secondary effect, which increases G_m_ with further increases in genipin concentration. The fit of Eq. 4, which is the sum of two logistic functions, reveals that the EC_50_ is 97 ± 19 *μ*M for the inhibitory effect of genipin and 351 ± 32 *μ*M for the genipin-mediated G_m_ increase. In contrast, geniposide showed only an inhibitory effect with EC50 = 693 ± 90 *μ*M.

To assess the origin of the G_m_ increase, we studied H^+^ permeability of the membrane by imposing a pH gradient on BLM with reconstituted UCP1 in the presence of 50 *μ*M and 1 mM genipin. According to the Nernst theory, a transmembrane pH gradient (*Δ*pH = 0.4 in our experiments) leads to a shift of current-voltage curves, *Δ*U (Fig. 6 *B*). In the presence of 50 *μ*M genipin, the measured *Δ*U = 24.4 ± 1.4 mV (Fig. 6 *B*, *inset*, *first bar*) was similar to the theoretical value (*Δ*U_Nernst_ = 23.6 mV). In contrast, 1 mM genipin reduced the shift to *Δ*U = 8.5 ± 0.5 mV (Fig. 6 *B*, *inset*, *second bar*), which pointed to a decrease in the proton selectivity of the membrane. The latter can be ascribed to the appearance of the additional conductance for sodium ions, which was the only cation present in the buffer.

### UCP1 is required for genipin-induced membrane defects

**Figure 7:**
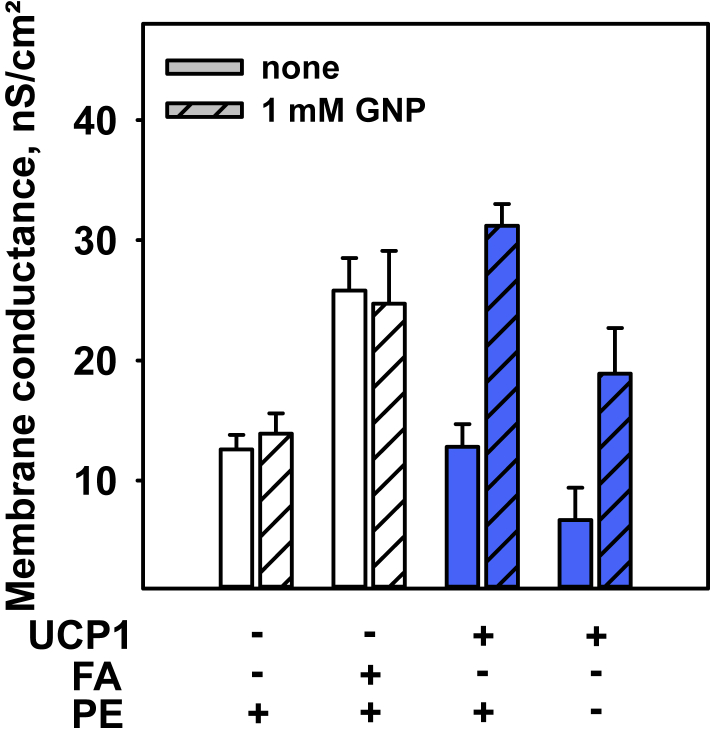
Genipin-UCP1 interaction leads to an increase in membrane conductance. Shown is the total membrane conductance of different membranes in the presence and absence of 1 mM GNP with membranes containing 45:45:10 mol% phosphocholine (PC):PE:CL (*first set*), 45:45:10 mol% PC:PE:CL, 15 mol% AA (*second set*), 45:45:10 mol% PC:PE:CL, 4 *μ*g/(mg lipid) UCP1 (*third set*), 90:10 mol% PC:CL, and 4 *μ*g/(mg lipid) UCP1 (*fourth set*). The lipid concentration was 1.5 mg/mL for all measurements. The buffer solution contained 50 mM Na_2_SO_4_, 10 mM Tris, 10 mM MES, and 0.6 mM EGTA at pH = 7.34 and T = 33°C. The data are the means ± SD of three independent measurements. To see this figure in color, go online.

Because genipin was shown to covalently bind to primary amines, we next investigated whether genipin modifies lipids and/or UCP1. We applied 1 mM genipin to membranes containing free FAs, UCP1, and DOPE in different combinations (Fig. 7). In the absence of UCP1, genipin did not alter the total membrane conductance of membranes independent of the lipid composition and the presence of FAs (Fig. 7, first and second data set, Fig. S5). In the presence of UCP1, the conductance increases from 12.8 ± 1.9 to 31.2 ± 1.8 nS/cm^2^ (Fig. 7, third data set) independently of the DOPE content of the membrane (Fig. 7, fourth data set).

### Genipin covalently binds to DOPE

**Figure 8:**
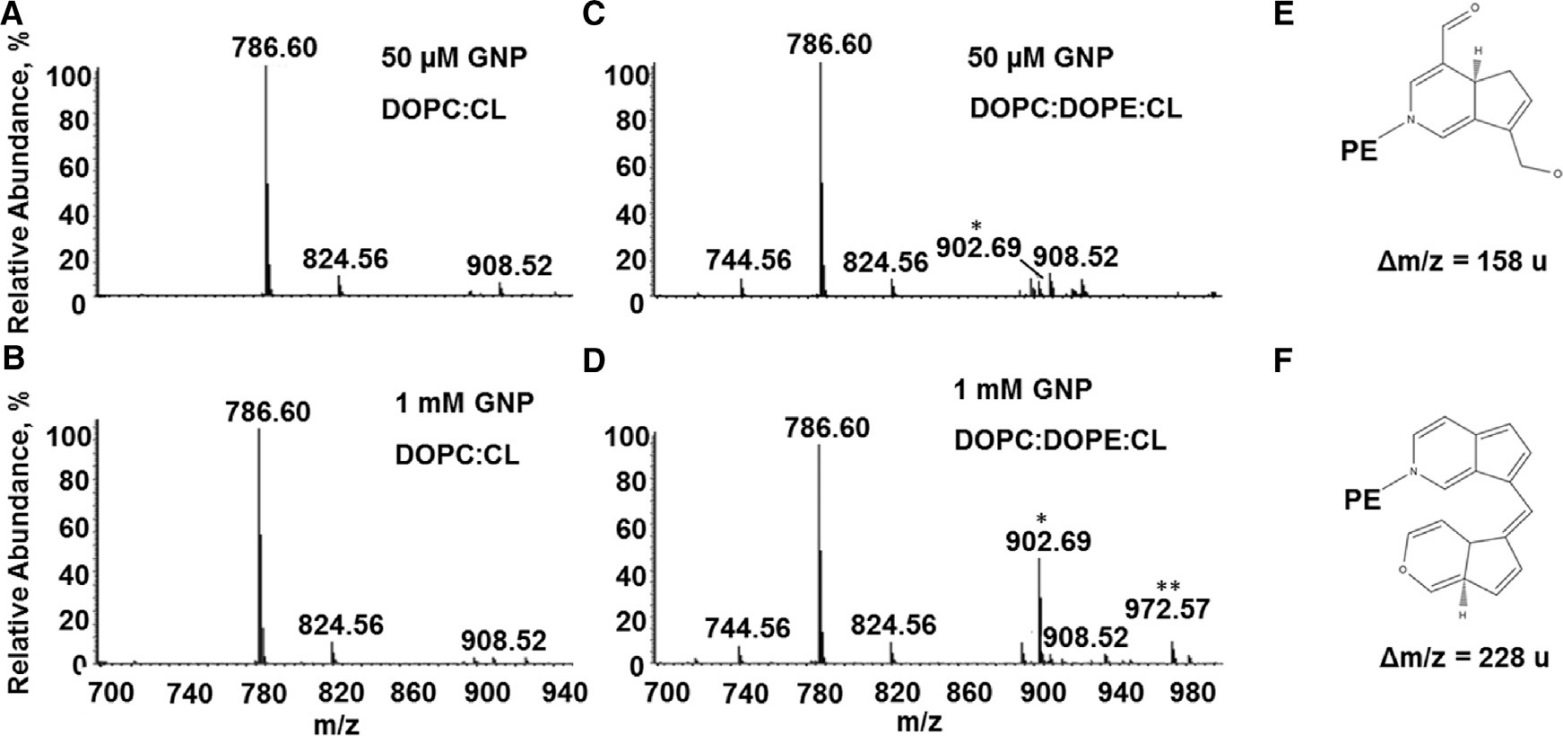
Mass spectra of genipin-modified lipids. Shown are the ESI-Orbitrap spectra of DOPC:CL (*A* and *B*) and DOPC:DOPE:CL (*C* and *D*) liposomes incubated with 50 *μ*M (*A* and *C*) or 1 mM (*B* and *D*) genipin. The signals at *m/z* 744.56, 786.60, and 824.56 correspond to protonated DOPE, DOPC, and potassium adducts of DOPC, respectively. The signals that have asterisks (*m/z* 902.69 and 972.57) correspond to DOPE-genipin adducts with mass increments of 158 (*E*) and 228 amu (*F*), respectively.

Direct infusion mass spectrometry (MS) analysis of liposomes in the absence of UCP1 revealed that genipin is covalently bound to a phosphoethanolamine (PE) headgroup at a concentration of 50 *μ*M (Fig. 8 *C*; ion at *m/z* 902.69) and 1 mM (Fig. 8 *D*; ions at *m/z* 902.69 and 972.57) but does not modify DOPC:CL liposomes (Fig. 8, *A* and *B*). Two adducts with DOPE primary amino groups were detected corresponding to the mass increments of 158 and 228 atomic mass units (amu) (Fig. 8, *E* and *F*).

### Genipin modifies lysine and/or cysteine in UCP1 in a concentration-dependent manner

**Figure 9:**
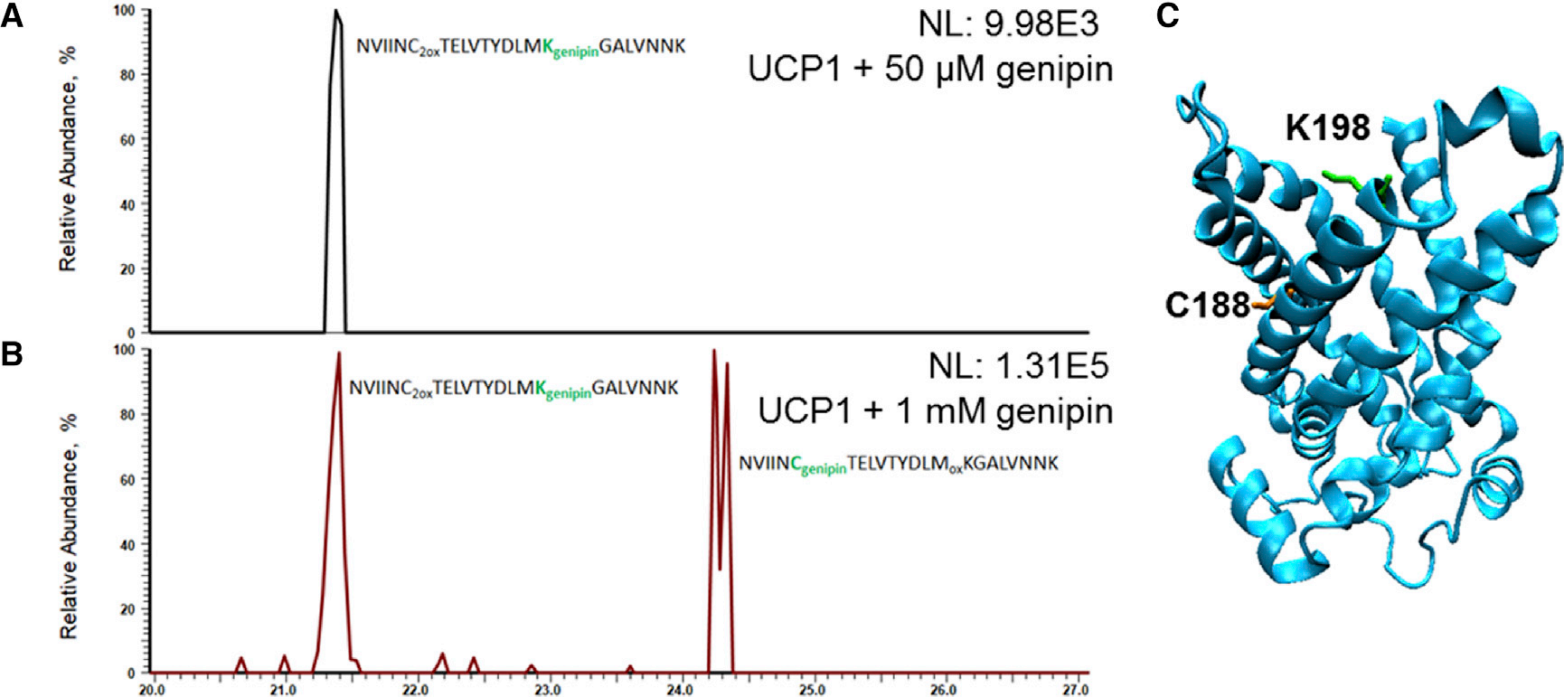
Extracted ion chromatograms of genipin-modified UCP1 tryptic peptides. Signals at *m/z* 919.47 and 914.14 correspond to UCP1 tryptic peptide ^184^NVIICTELVTYDLMKGALVNNK^206^ carrying K198 (919.47; tR = 21.4) and C188 (919.14; tR = 24.2), respectively, modified with genipin adduct of 158 amu after incubation with 50 *μ*M (*A*) and 1 mM (*B*) genipin. Corresponding CID spectra are provided in Fig. S1. (*C*) Positions of cysteine C188 and lysine K198 in UCP1, at which genipin modifications were detected. Three-dimensional structure of UCP1 was computed based on the crystallographic structure of ANT (PDB: 1OKC) using PyMol. To see this figure in color, go online.

Similar to PE amino groups, genipin can form covalent adducts with nucleophilic amino acid residues in proteins. To identify genipin targets in UCP1, protein reconstituted in liposomes was incubated with 50 *μ*M and 1 mM of genipin, separated by SDS-PAGE, digested with trypsin, and analyzed by liquid chromatography-tandem MS. Using mass increments identified as genipin-specific adducts on PE (see Genipin covalently binds to DOPE) as variable modifications during a database search, we identified tryptic peptide ^184^NVIICTELVTYDLMKGALVNNK^206^ carrying K198 (signal at *m/z* 919.47^3+^) and C188 (signal at *m/z* 919.14^3+^) modified with a genipin adduct of 158 amu (Fig. 9). Lysine-modified peptide (Fig. 9 *A*) was detected in UCP1 samples incubated with both 50 *μ*M and 1 mM genipin, whereas cysteine modification was present only in the sample incubated with 1 mM genipin (Fig. 9 *B*). CID-tandem mass spectra confirming the assignment of modification sites are shown in Fig. S6.

### Genipin at low concentrations inhibits UCP1 by binding to arginines

**Figure 10:**
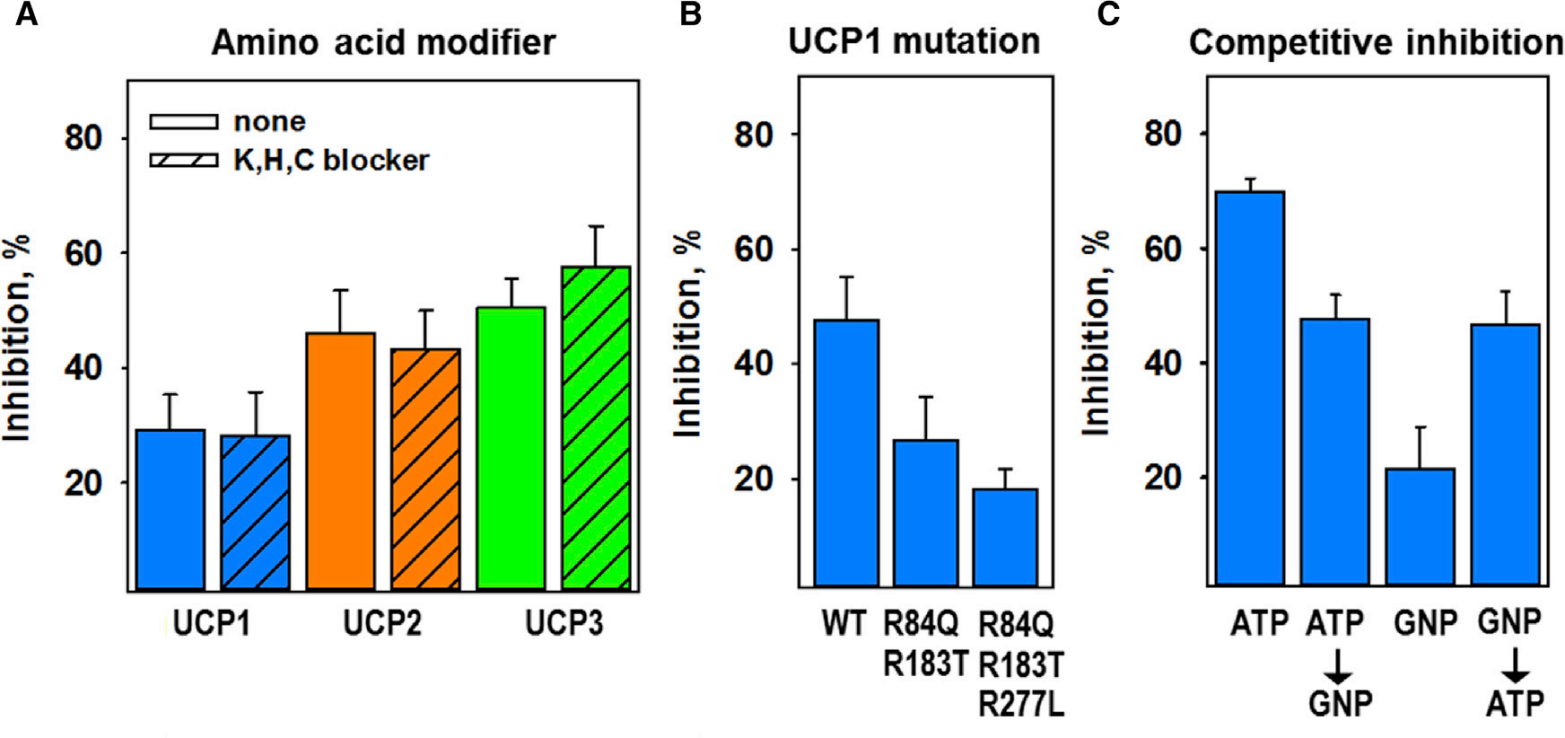
Electrophysiological analysis of the amino acids of UCP1 involved in the inhibition mechanism by genipin. (*A*) Effect of 50 *μ*M genipin on UCP1, UCP2, and UCP3 pretreated with NHS, MNBS, and NEM (lysine, histidine, and cysteine blockers, respectively). Membranes were made of 45:45:10 mol% DOPC:DOPE:CL and reconstituted with 15 mol% AA. The protein concentration was 5–6.5 *μ*g/(mg lipid). The concentration of NHS was 1.3 *μ*M for UCP1 and 1.1 *μ*M for UCP2 and UCP3, that of MNBS was 0.2 *μ*M for UCP1 and UCP2 and 0.3 *μ*M for UCP3, and that of NEM was 0.5 *μ*M for UCP1 and UCP3 and 0.3 *μ*M for UCP2 and UCP3, all according to the number of lysine, histidine, and cysteine residues per protein. The left bar of each pair is the control comprising unmodified proteins with the addition of a similar amount of water to the buffer. (*B*) Inhibition of UCP1wt (4 *μ*g/(mg lipid)), UCP1R84Q/R183T (5 *μ*g/(mg lipid)), and UCP1R84Q/R183T/R277L (4 *μ*g/(mg lipid)) by 100 *μ*M genipin, which was directly added to the buffer solution. (*C*) Competitive inhibition of UCP1 with genipin (GNP) and ATP. The first bar is the inhibition after the addition of ATP, the second bar is the inhibition by genipin added after ATP, the third bar is the inhibition by genipin, and the fourth bar is the inhibition by ATP added after genipin. The concentrations of ATP and genipin were 3 mM and 50 *μ*M, respectively. The protein concentration was 6 *μ*g/(mg lipid). The membranes were made of 45:45:15 mol% DOPC:DOPE:CL reconstituted with 15 mol% AA, and the lipid concentration was 1.5 mg/mL. The buffer solution consisted of 50 mM Na_2_SO_4_, 10 mM Tris, 10 mM MES, and 0.6 mM EGTA at pH = 7.34 and T = 33°C. The results are the means ± SD of three independent experiments. To see this figure in color, go online.

Further, we used NHS, MNBS, and NEM to block the lysine, histidine, and cysteine residues of UCP1, respectively (51). As the unmodified control, UCP1 was incubated with an equal volume of water. The inhibition of UCPs by 50 *μ*M genipin was not significantly different in the presence (second bar of each data set) or absence (first bar of each data set) of the amino acid blockers (Fig. 10 *A*).

Arginines 84, 183, and 277 were shown to be crucial for the inhibition of UCP1 by purine nucleotides (39,52). We used double (UCP1R84Q/R183T) and triple (UCP1R84Q/R183T/R277L) UCP1 mutants to test whether these arginines may also be important for genipin action. The administration of 100 *μ*M genipin showed a decreased inhibitory effect of genipin on the UCP1 double (Fig. 10 *B*, *second bar*) and triple mutant (26.8 ± 7.5% and 19.2 ± 3.5%, respectively, Fig. 10 *B*, *third bar*) compared to the wt (48.6 ± 7.5%, Fig. 10 *B*; *first bar*).

Finally, we tested whether the inhibition of UCP1 by ATP and genipin is competitive. Fig. 10 *C* shows that the total membrane conductance was similar in the presence of both substrates (52.2 ± 4.1%, second bar and 53.3 ± 5.8%, fourth bar relative to the uninhibited control), regardless of the order in which the inhibitors were added (first ATP and second genipin (Fig. 10 *C*; *first two bars*) or first genipin and second ATP (Fig. 10 *C*; *last two bars*)).

## Discussion

### Genipin influenced the activity of membrane proteins differently

In a prior study, the effect of genipin on UCP2 was compared in kidney mitochondria isolated from wt and UCP2-deficient mice (18). Genipin inhibited the proton leak produced by xanthine/xanthine oxidase or by hydroxynonenal only in mitochondria from wt mice (Figs. 1 and 2; (18)). In UCP2-deficient mice, no proton leak was recorded. This effect is difficult to explain because the presence of UCP2 at the protein level was not demonstrated in the mentioned study, and no UCP2 was found in the kidney previously (23,53). The results presented in this work support the view that UCP2 in not the only target of genipin as it also inhibits the proton transport mediated by UCP1 and UCP3 reconstituted in the planar bilayer membranes. Furthermore, it decreases the activity of isolated CIII and activates DIC in isolated mitochondria. Because genipin interacts with multiple mitochondrial proteins, caution when interpreting the data obtained from cells and isolated mitochondria is warranted.

The conclusion about the action of genipin on UCP2 is usually based on the measurements of mitochondrial transmembrane potential, *Φ*_m_. An evaluation of the literature reveals that the obtained results are controversial. One of the possible reasons can be that in many studies, UCP2 is artificially expressed in cells in which it is normally absent. Our group and others have recently shown that UCP2 is confined to rapidly proliferating cells that rely on glycolysis, such as stem cells, activated immunological cells, and cancer cells (22,28,53). The expression of UCP2 in cells that have other metabolic pathways can seriously affect the physiological function of UCP2. Genipin decreases *Φ*_m_ in a concentration-dependent manner if added to neuroblastoma (N18TG2) cells, which naturally express UCP2 (Fig. 1; (28)). This finding contradicts the idea that genipin affects only UCP2 because its inhibition would cause the increase of *Φ*_m_. The drop in *Φ*_m_ could be rather explained by the inhibition of respiratory chain proteins, for example, CIII, as shown in this study (Fig. 3). Our study did not confirm the inhibition of UCP2 expression by genipin as reported for the diabetic kidney (9).

### The inhibition mechanism of UCPs by genipin at low concentrations is similar to that of purine nucleotides

Genipin is typically used at a concentration of 1–250 *μ*M to demonstrate its effects on UCP2 (18,54) because higher concentrations lead to cross-linking. However, no comprehensive molecular mechanism for UCP2 inhibition has been proposed until now. We have chosen UCP1 as a model protein for the investigation of the molecular mechanism of the concentration-dependent effect of genipin because of the following: 1) its activation and inhibition mechanisms are well studied (39), and 2) the high homology between both proteins allows us to extend the obtained results to UCP2. We observed qualitatively different behavior of G_m_ at low and high genipin concentrations, as follows: 1) decrease of G_m_ at concentrations below 200 *μ*M, which corresponds to the inhibition of UCP1-mediated proton transport and 2) increase of unspecific G_m_ at concentrations above 200 *μ*M (Fig. 6 *A*). Using MS, we revealed three target sites for the interaction between genipin and protein/lipid. Genipin forms adducts with 1) K198 of UCP1 2), C188 of UCP1, and 3) the amino group of DOPE. Interestingly, we did not observe differences in G_m_ compared to the unmodified control when the lysines, histidines, or cysteines of UCP1 were blocked (Fig. 10 *A*). This supports our previous results that modification of these amino acids in UCP1 does not affect its activity (51,55).

**Figure 11:**
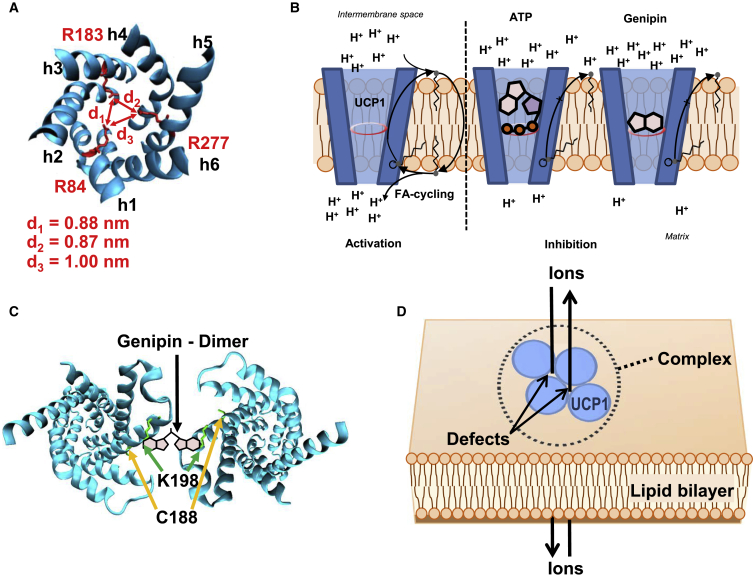
Proposed mechanisms of the genipin-UCP1 interaction. (*A*) Position of the three arginines (R84, R183, and R277) inside the cavity of UCP1, which are substantial for its inhibition by purine nucleotides and genipin. (*B*) Mechanism of UCP1 inhibition by genipin at low concentrations. Left: according to the FA cycling model, protonated FA flips back to the matrix and releases the proton, which decreases the transmembrane potential. UCP1 facilitates the transport of FA anions from the matrix to the intermembrane space (IMS). Middle: the phosphate groups of purine nucleotides (here shown as a sketch of ATP) interact with R84, R183, and R277, which inhibit the transport of FAs to the IMS. Right: similarly to PNs, genipin interacts with arginines, thereby preventing the transport of FAs to the IMS. (*C*) Position of C188 and K198 in UCP1 at which genipin modifications were detected. The structure of UCP1 was computed based on the crystallographic structure of ANT (PDB: 1OKC). Putative three-dimensional structure of UCP1 was visualized using PyMol. (*D*) Cross-linking mechanism: UCP1 (*blue circles*) molecules are cross-linked by genipin, which leads to the formation of protein complexes (indicated by the *dotted circle*). Defects, which appear within the complexes, allow the nonspecific transport of ions, thereby increasing total G_m_. To see this figure in color, go online.

In contrast, the mutation of three distinct arginines, R84, R183, and R277, which are located within the cavity of UCP1 (39), turned out to be crucial for genipin-mediated inhibition (Fig. 11, *A* and *B*). The same arginines were shown to be involved in the inhibition of UCP1 by purine nucleotides (39,52,56). The decreased inhibition of proton transport in arginine mutants together with the competitive inhibition between genipin and ATP suggests a similar molecular mechanism by which genipin and purine nucleotides inhibit UCPs. Indirect support for this mechanism comes from the fact that genipin failed to inhibit a pore-forming toxin *α*HL. In contrast to UCPs and CIII, the transmembrane domain of *α*HL is a funnel made of *β*-sheets (57), which allows nonspecific exchange of water and molecules up to ∼2000 Da (58). Thus, we assumed that genipin is translocated through the pore without interaction.

Measurements of G at a transmembrane pH gradient revealed that genipin at a concentration of more than 400 *μ*M promoted nonspecific ion leak through the membrane. This G_m_ increase was neither lipid nor FA dependent and was only observed in the presence of UCPs. Based on literature data (17), we propose that UCPs form clusters by cross-linking in the presence of genipin, which leads to pore formation within the membrane (Fig. 11, *C* and *D*). The cross-linking reaction is usually characterized by a nucleophilic attack of primary amines at carbon 3, which results in the opening of the dihydropyran ring of genipin (59,60). In a second step, the oxygen is replaced by the nitrogen of the primary amine that binds genipin covalently to biomolecules like proteins. In this state, genipin polymerizes and links two binding sites within a molecule or two distinct molecules. In the case of UCPs, two genipin molecules obviously links proteins via K198 and C188, as proposed in Fig. 11 *C*. Modified proteins allow nonspecific transmembrane ion transport (Fig. 11 *D*) and, as a consequence, an increase in the total G_m_. The lack of an inhibitory effect of geniposide (Fig. 6 *A*), which has no cross-linking ability, supports this mechanism.

## Conclusions

Our results imply that the effects of genipin at the cellular and mitochondrial levels cannot be associated with the selective inhibition of UCP2 because, aside from the inhibition of UCP-mediated proton transport, genipin influences the activities of other mitochondrial proteins, such as CIII and DIC. Three arginines are key in the inhibition of UCPs by genipin and demonstrate that the molecular mechanisms of UCP inhibition by genipin and purine nucleotides might be similar. Our results contribute to the understanding of the multiple effects of genipin in vivo and will serve as a basis for the development of effective drugs suitable for treatment of cancer, inflammation, and diabetes.

## Author Contributions

J.K. performed experiments, analyzed data, and wrote the manuscript. A.R., L.Z., M.M., T.I.R., Y.N.A., L.G., and M.F. performed experiments, analyzed data, and partly described the results for the manuscript. E.E.P. designed the research, supervised the project, and wrote the final manuscript. All authors edited and approved the final article.

## Supporting Material

Document S1. Figs. S1–S6

Document S2. Article plus Supporting Material
