# Supplementary material for: Molecular Mechanisms Responsible for Pharmacological Effects of Genipin on Mitochondrial Proteins
Source: Biophys J. 2019 Oct 24;117(10):1845–57. doi: 10.1016/j.bpj.2019.10.021 (PMC7031773; doi:10.1016/j.bpj.2019.10.021)
Supplement: Document S1. Figs. S1–S6 [file mmc1.pdf]

Biophysical Journal, Volume *117*

Supplemental Information

Molecular Mechanisms Responsible for Pharmacological Effects of Genipin on Mitochondrial Proteins

Jürgen Kreiter, Anne Rupprecht, Lars Zimmermann, Michael Moschinger, Tatyana I. Rokitskaya, Yuri N. Antonenko, Lars Gille, Maria Fedorova, and Elena E. Pohl

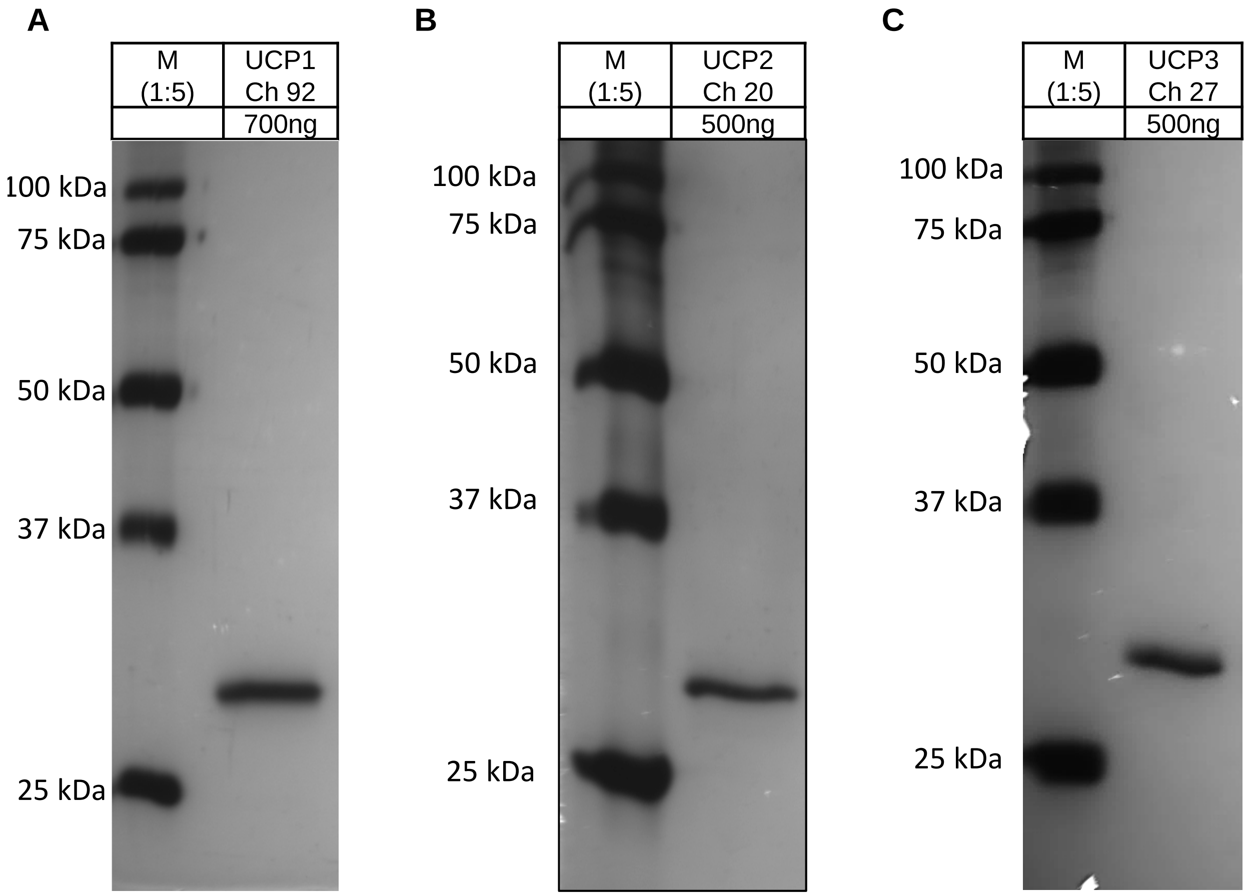

Fig. S1. Silverstaining of proteoliposomes, containing recombinant UCP1 (A), or UCP2 (B), or UCP3 (C) and loaded on a 15% SDS-gel.

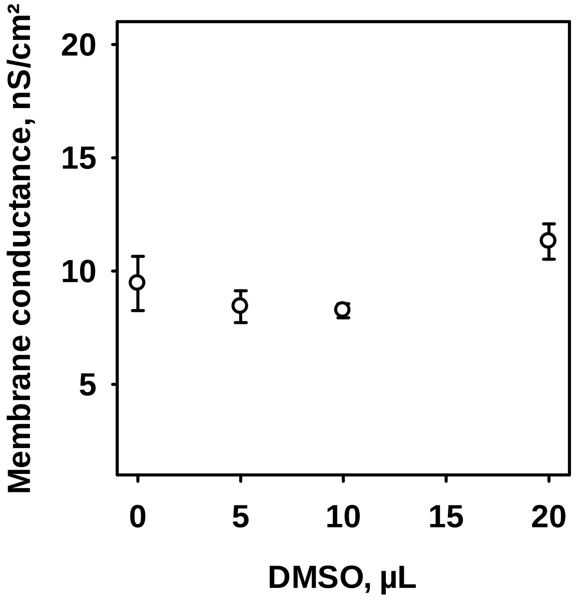

Fig. S2. Effect of DMSO on lipid membrane conductance.

Lipid bilayer membranes were made of 45:45:10 mol% DOPC:DOPE:CL with a lipid concentration of 1.5 mg/ml. Buffer contained 50 mM Na_2SO_4, 10 mM Tris, 10 mM MES and 0.6 mM EGTA at pH = 7.34 and T = 33°C.

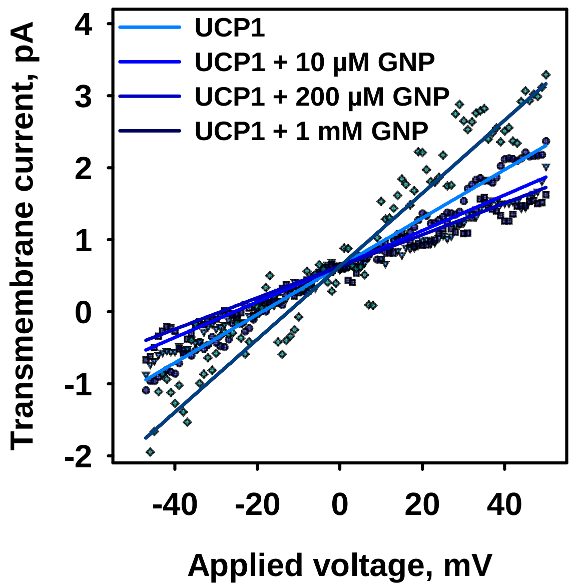

Fig. S3. Representative current-voltage recordings of lipid membranes reconstituted with UCP1 in the presence of 0, 10, 200 and 1000 µM genipin (GNP).

Protein and lipid concentrations were 4 µg/mg of lipid and 1.5 mg/ml, respectively. Bilayer membrane was made of 45:45:10 mol% DOPC:DOPE:CL, reconstituted with 15 mol% AA. Buffer contained 50 mM Na_2SO_4, 10 mM Tris, 10 mM MES and 0.6 mM EGTA at pH = 7.34 and T = 33°C. Slopes of the linear fits give the total membrane conductance.

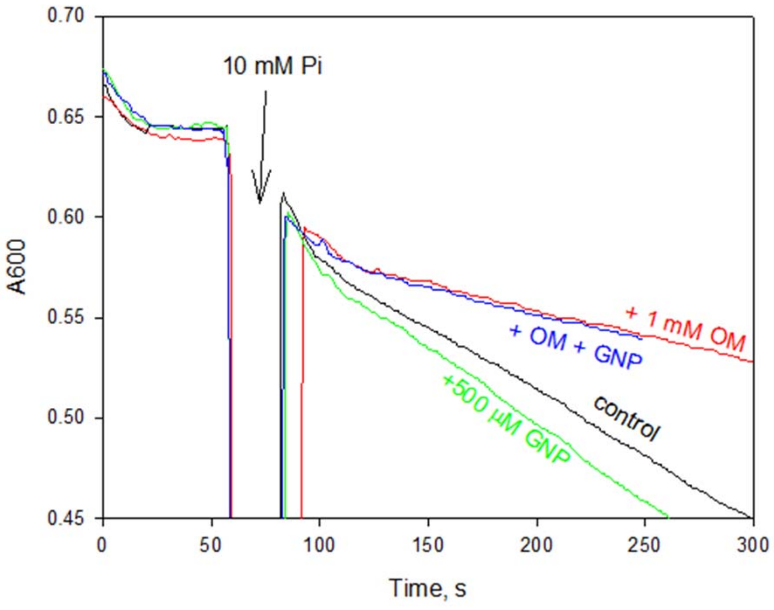

Fig. S4. Control experiments to Fig. 4, A showing the specificity of genipin (GNP) on DIC activity. For the experimental conditions see the legend to Fig. 4, A. OM – octyl – malonate.

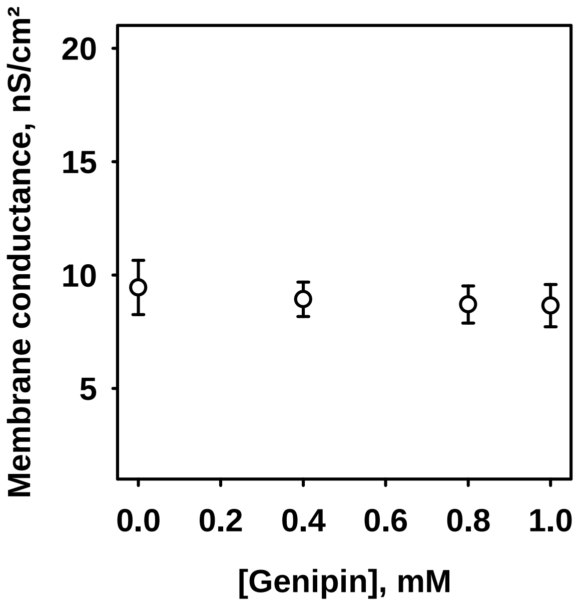

Fig. S5. Effect of genipin on conductance of lipid bilayer membranes

Bilayer membrane was made of 45:45:10 mol% DOPC:DOPE:CL with a lipid concentration of 1.5 mg/ml. Buffer contained 50 mM Na_2SO_4, 10 mM Tris, 10 mM MES and 0.6 mM EGTA at pH = 7.34 and T = 33°C.

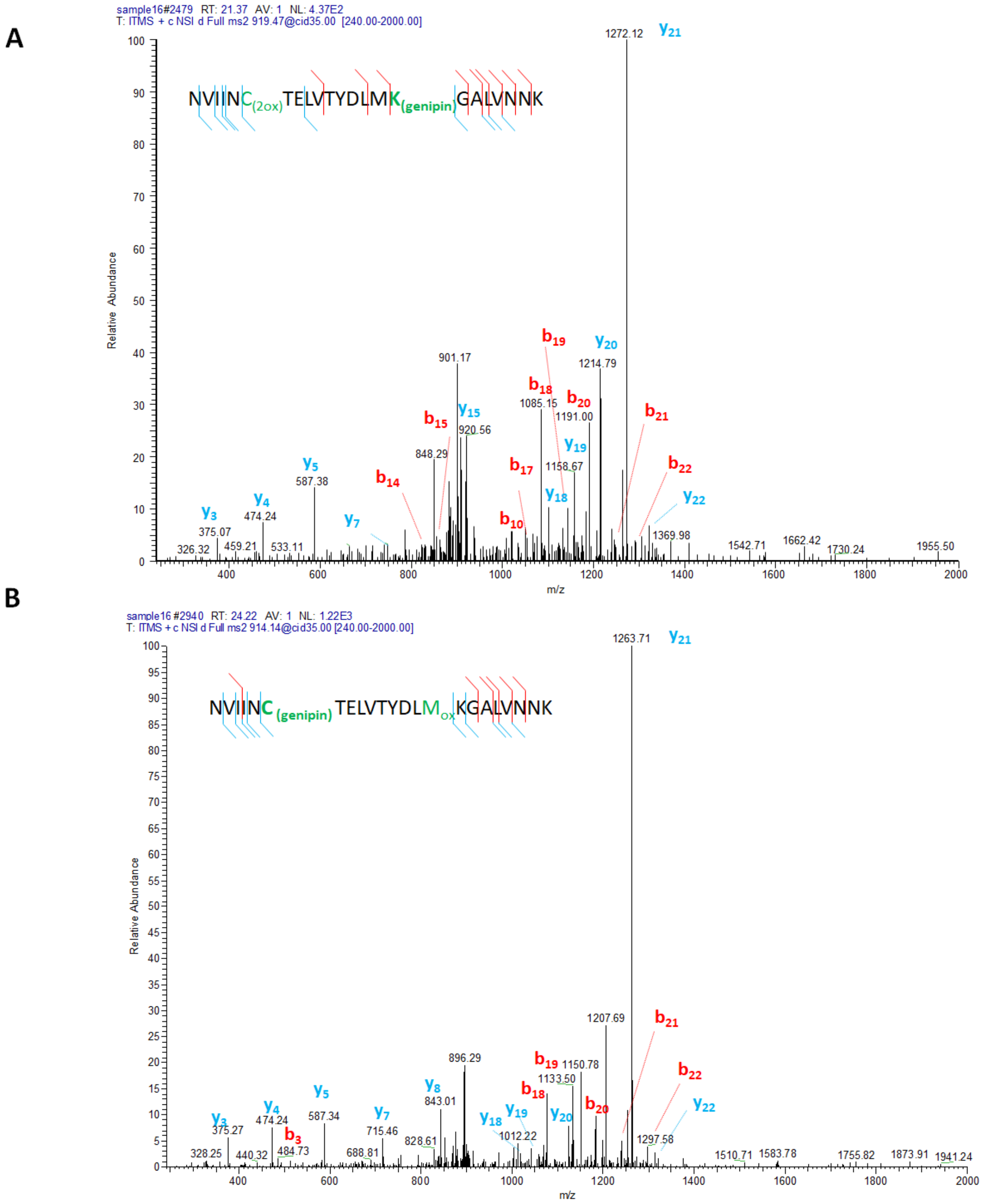

Fig. S6. CID tandem mass spectra of genipin modified UCP1 tryptic peptides.
